# Neuropeptide Y deficiency perturbs L-type calcium and outward potassium currents leading to ventricular arrhythmogenesis in mice

**DOI:** 10.1172/JCI204258

**Published:** 2026-08-11

**Authors:** Seyi E. Elasoru, Christian Miccile, Jingwu Pan, Zhaoyang Zhang, Kalyanam Shivkumar, Herbert Herzog, Robert D. Harvey, Zhilin Qu, Olujimi A. Ajijola

**Affiliations:** 1 Department of Medicine-Cardiology, UCLA, Los Angeles, California, USA.; 2 Department of Physics, School of Physical Science and Technology, Ningbo University, Ningbo, Zhejiang, China.; 3 St. Vincent’s Centre for Applied Medical Research, Darlinghurst, New South Wales, Australia.; 4 Department of Pharmacology, University of Nevada, Reno, Nevada, USA.

**Keywords:** Cardiology, Neuroscience, Arrhythmias, Mouse models

## Abstract

Elasoru and colleagues present findings revealing a novel role for Neuropeptide Y (a sympathetic co-transmitter) in cardiomyocyte ion channel regulation and action potential duration.

**To the Editor:** Neuropeptide Y (NPY), the most abundant neuropeptide in the heart and cotransmitter with norepinephrine, has established roles in cardiovascular physiology and may contribute to sustained cardiac adrenergic activation because of its prolonged biological activity (1). However, its effects on cardiac electrophysiology are incompletely understood. To assess this, we generated mice with complete (homozygous) and partial (heterozygous) NPY loss (NPY^–/–^ and NPY^–/+^) using homologous recombination in embryonic stem cells (2).

Our study examined 8- to 12-week-old male and female mice, and similar findings are reported for both sexes. Echocardiography revealed no differences in cardiac structure or systolic function between the NPY^–/–^ and NPY^–/+^ (control) groups (Figure 1, A–C). Heart weight (HW) and the HW/BW ratio were likewise unchanged (Figure 1, D and E). However, electrocardiography revealed striking electrical abnormalities in NPY^–/–^ mice (Figure 1F), including a lower heart rate (496 ± 38 vs. 544 ± 39 bpm, *P* < 0.05; Figure 1G) and a prolonged QT interval (42 ± 5 vs. 27 ± 4 ms, *P* < 0.0001; Figure 1H). These results demonstrate that genetic depletion of NPY selectively impaired cardiac electrical activity, particularly ventricular repolarization, without affecting the overall structure and systolic performance.

To further confirm the impaired ventricular repolarization, we measured the action potential (AP) in ventricular cardiomyocytes isolated from NPY^–/–^, NPY^–/+^, and WT mice. NPY^–/–^ cardiomyocytes exhibited significantly prolonged AP duration (APD) compared with control cardiomyocytes (111 ± 28 vs. 42 ± 11 vs. 40 ± 21 ms, *P* < 0.001; Figure 1, I–K), whereas the resting membrane potential (RMP) was unchanged (Figure 1L), suggesting intrinsic remodeling of repolarizing ion channel function. Interestingly, exogenous NPY (100 nM) significantly (*P* < 0.001) shortened the APD measured at 90% repolarization (APD_90_) in NPY^–/–^ cardiomyocytes, producing a greater reduction than in control cardiomyocytes (32 vs. 15 vs. 18 %; Figure 1, M and N). These results suggest that endogenous NPY was required for normal ventricular AP repolarization and prompted our investigation of the underlying ionic mechanisms.

Hence, we measured the L-type calcium current (I_Ca,L_) and the total outward K current (I_K,total_) in ventricular cardiomyocytes from NPY^–/–^ and control mice. Subsequently, we isolated the transient outward K current (I_to_), the slowly inactivating K current (I_K,slow_), and the steady-state K current (I_ss_) from I_K,total_ (Supplemental Figure 1, A–H) using established biophysical and pharmacological protocols (3–5). NPY depletion significantly (*P* < 0.01) increased peak I_Ca,L_ density across multiple test potentials (–20 to +30 mV) compared with the control (Figure 1, O and P). Among the 3 major repolarizing outward K currents, I_to_ was unchanged (Figure 1, Q and R), whereas I_K,slow_ and I_ss_ were significantly reduced in NPY^–/–^ cardiomyocytes compared with the control (8 ± 3 vs. 16 ± 5 pA/pF and 8 ± 4 vs. 15 ± 4 pA/pF, respectively; *P* < 0.001; Figure 1, S–V). These changes in I_Ca,L_, I_K,slow_, and I_ss_ likely underlie the prolonged APD in NPY^–/–^ cardiomyocytes.

To determine whether the observed changes in I_Ca,L_, I_K,slow_, and I_ss_ were sufficient to explain the electrophysiological phenotype of the NPY^–/–^ mice, we performed in silico simulations. Incorporating the experimentally measured changes in I_Ca,L_, I_K,slow_, and I_ss_ into a mouse ventricular AP model accurately reproduced the AP morphology observed in NPY^–/–^ cardiomyocytes (Supplemental Figure 2, A–D; supplemental material available online with this article; https://doi.org/10.1172/JCI204258DS1 Mechanistically, reduced I_K,slow_ enhanced peak I_Ca,L_ during phase 1 repolarization, increasing intracellular Ca [Ca]_i_ and the sodium-calcium exchange current (I_NCX_), thereby prolonging the APD (Supplemental Figure 2, E and F). Also, in human ventricular AP models (6), a 40% increase in I_Ca,L_ prolonged the APD, primarily through the direct effects of I_Ca,L_ on membrane voltage rather than changes in [Ca]_i_ and I_NCX_, supporting the translational relevance of our findings (Supplemental Figure 2G).

Consistent with the prolonged repolarization phenotype, NPY^–/–^ mice exhibited increased susceptibility to ventricular ectopy during β-adrenergic stimulation (Supplemental Figure 3, A–E), but these anomalies are independent of Y1 receptor (Y1R) signaling (Supplemental Figure 4, A–E).

In conclusion, this study reveals a congenital effect of NPY on ion channel function, the development of AP repolarization properties, and the QT interval. Additional studies are needed to identify the regulatory networks through which NPY depletion alters cardiac electrophysiology and to assess the implications of variable NPY expression on the human QT interval.

## Conflict of interest

OAA reports receiving honoraria from Medtronic, J&J Medtech/Biosense Webster, Biotronik, Abbott, and Boston Scientific. KS and OAA report owning stock in Anumana and nference. OAA and KS are co-founders of Neufera. KS is a consultant to Anumana and Boston Scientific.

## Funding support

This work is the result of NIH funding, in whole or in part, and is subject to the NIH Public Access Policy. Through acceptance of this federal funding, the NIH has been given a right to make the work publicly available in PubMed Central.

- NIH grant R01HL162717.
- Chan Zuckerberg Science Leadership Award (to OAA).
- NIH grant R01HL175074 (to ZQ and OAA).
- NIH grant P01HL164311 (to KS, RH, and OAA).

## Supplementary Material

Supplemental data

Supporting data values

## Figures and Tables

**Figure 1:**
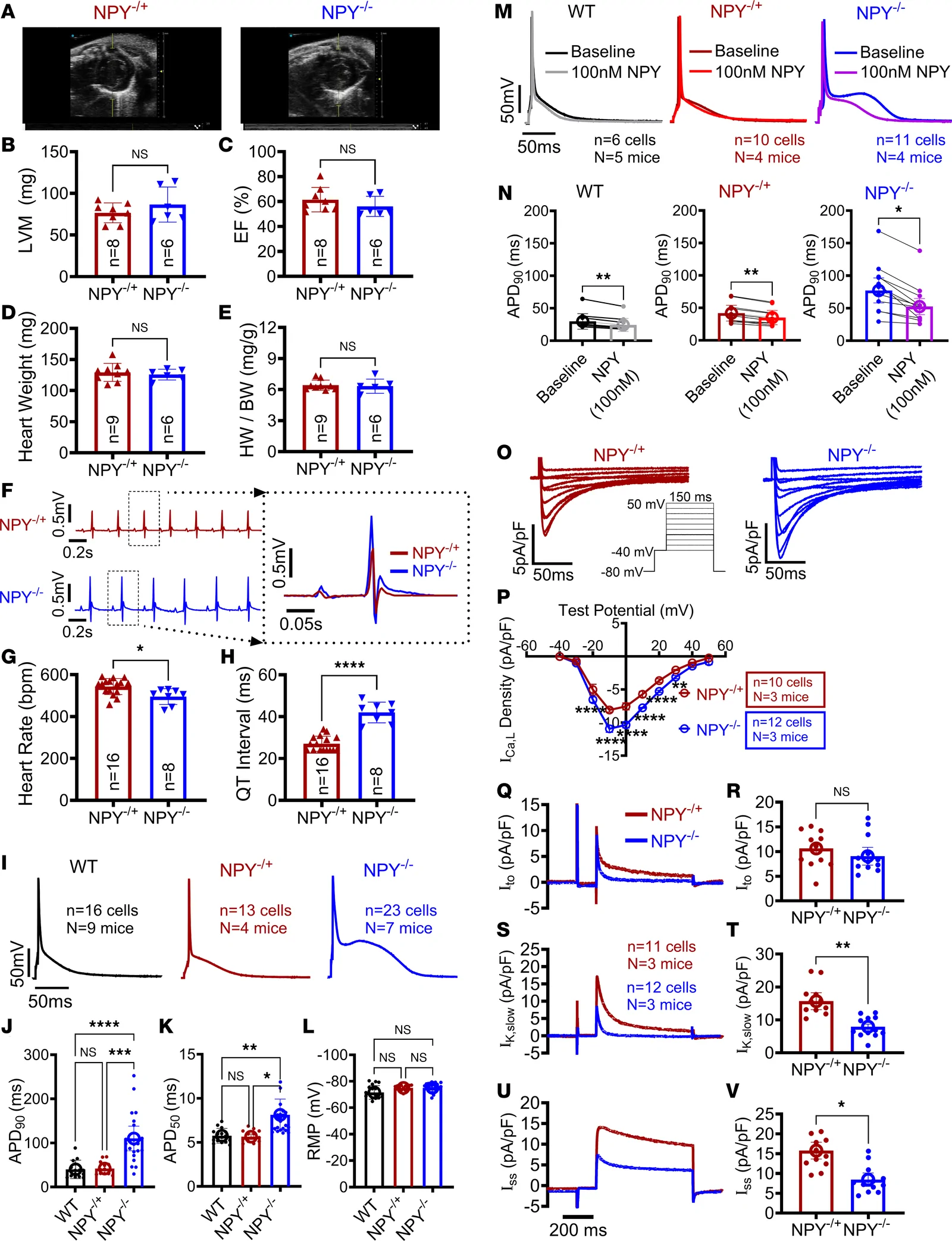
Congenital depletion of neuropeptide Y prolongs ventricular APD by discordant alterations in L-type calcium, delayed rectifier potassium, and steady-state potassium currents. (**A**) Representative echocardiography images of NPY^–/+^ and NPY^–/–^ mice. (**B**) Cardiac left ventricular mass (LVM). (**C**) Ventricular ejection fraction (EF) percentage. (**D**) HW. (**E**) HW/BW ratio. (**F**) Representative electrocardiogram recorded in NPY^–/+^ and NPY^–/–^ mice. (**G**) Heart rate (HR). (**H**) QT interval. (**I**) Representative AP recorded in WT, NPY^–/+^, and NPY^–/–^ cardiomyocytes. (**J**) APD_90_. (**K**) AP duration measured at 50% repolarization (APD_50_). (**L**) RMP. (**M**) Superimposed representative APs recorded at baseline and after exogenous NPY (100 nM) application. (**N**) APD_90_ at baseline and after exogenous NPY application in each group. (**O**) Representative I_Ca,L_ traces recorded in NPY^–/+^ and NPY^–/–^ cardiomyocytes. (**P**) Current-voltage relationship plot of I_Ca,L_. (**Q**) Representative I_to_ traces recorded in NPY^–/+^ and NPY^–/–^ cardiomyocytes. (**R**) I_to_ density. (**S**) Representative I_K,slow_ traces recorded in NPY^–/+^ and NPY^–/–^ cardiomyocytes. (**T**) I_K,slow_ density. (**U**) Representative I_ss_ traces recorded in NPY^–/+^ and NPY^–/–^ cardiomyocytes. (**V**) I_ss_ density. Currents were normalized to cell capacitance and are expressed as current density (pA/pF). Data are shown as the mean ± SD, with cardiomyocyte data nested within mice. *P < 0.05, **P < 0.01, ***P < 0.001, and ****P < 0.0001. Comparisons between two groups were performed using 2-tailed nested *t* test, Student’s *t* test, or the Mann-Whitney test, as appropriate. Comparisons among multiple groups were performed using nested 1-way ANOVA, ordinary 2-way ANOVA, mixed-effects analysis, or the Kruskal-Wallis test, as appropriate.

## Version 1 08/11/2026

In-Press Preview

## Version 2 10/01/2026

Electronic publication
