# Supplementary material for: Neuropeptide Y deficiency perturbs L-type calcium and outward potassium currents leading to ventricular arrhythmogenesis in mice
Source: J Clin Invest. 2026 Aug 11;136(19):e204258. doi: 10.1172/JCI204258 (PMC13626841; doi:10.1172/JCI204258)
Supplement: Supplementary file 1 [file jci-136-204258-s198.pdf]

Neuropeptide Y deficiency perturbs L-type calcium and outward potassium currents leading to ventricular arrhythmogenesis in mice

Seyi E. Elasoru [a], Christian Miccile [a], Jingwu Pan [a], Zhaoyang Zhang [b], Kalyanam Shivkumar [a], Herbert Herzog [c], Robert Harvey [d], Zhilin Qu [a], Olujimi A. Ajijola [a].

a. Department of Medicine-Cardiology, University of California, Los Angeles, CA, USA
b. Department of Physics, School of Physical Science and Technology, Ningbo University, Ningbo, Zhejiang, China
c. St Vincent's Centre for Applied Medical Research, 405 Liverpool St. 2010 NSW Darlinghurst, Sydney Australia
d. Department of Pharmacology, University of Nevada, Reno, NV, USA

Funding Support and Author Disclosures: This work was supported by R01HL162717, a Chan Zuckerberg Science Leadership Award to OAA, R01HL175074 Award to ZQ & OAA, and P01HL164311 Award to KS, RH & OAA.

Relationships with Industry: Dr. Ajijola reports honoraria from Medtronic, J&J Medtech/Biosense Webster, Biotronik, Abbott and Boston Scientific. Drs. Shivkumar & Ajijola report stock in Anumana and nFerence. Drs. Ajijola & Shivkumar are co-founders of Neufera. Dr. Shivkumar is a consultant to Anumana and Boston Scientific. No other conflicts to report.

Running Title: Loss of NPY prolongs QT-interval and ventricular APD

Corresponding Author:

Olujimi Ajijola, MD, PhD

Department of Medicine-Cardiology

100 Medical Plaza, Suite 660

Los Angeles, CA 90095

Phone: 310-206-2235, Fax: 310-825-2092, E-mail: OAjijola@mednet.ucla.edu

Supplemental Methods

Sex as a Biological Variable

Both male and female mice (8-12 weeks old) were included in this study. Data from both sexes were evaluated, and because no sex-dependent differences were identified, the data were pooled for analysis unless otherwise indicated.

Experimental Animals

Animals were housed at UCLA Division of Laboratory Animal Medicine (DLAM) facilities, allowed free access to water and standard pellet diet, and maintained at ambient room temperature between 20 and 25°C, under a 12 h:12 h light/dark cycle. Wild-type mice (C57BL/6J) were obtained from the Jackson Laboratory. NPY knockout (KO) mice were received as a generous gift from Dr. Herbert Herzog's laboratory at St Vincent's Centre for Applied Medical Research, 405 Liverpool St. 2010 NSW Darlinghurst, Sydney Australia. Subsequently, the mice were bred in our laboratory to produce NPY heterozygous KO ($NPY^{-/+}$), NPY homozygous KO ($NPY^{-/-}$) and NPY wild-type ($NPY^{+/+}$) mice used for the study. Detailed strategy of how this genetic mouse model for NPY deficiency was generated using homologous recombination in embryonic stem cells has been described elsewhere (1). NPY Y1 receptor homozygous KO ($Y1R^{-/-}$) mice were also used in the study.

Echocardiography and Electrocardiography

Mice were rapidly anesthetized under 3% isoflurane, placed on a heated platform, then maintained under minimal (1.5%) isoflurane and transthoracic echocardiogram was recorded using a Vevo 3100 LT (FUJIFILM VisualSonics Inc., Toronto, Canada). Key indicators of ventricular integrity and systolic function including left ventricular mass (LVM), ejection fraction (EF), and fractional shortening (FS) were evaluated from M-mode images obtained in the parasternal short axis view. Likewise, electrocardiogram was recorded under similar conditions

using PowerLab 16/35 data acquisition system (ADInstruments, New Zealand) connected to a Bio Amp amplifier (ADInstruments, New Zealand). Briefly, needle electrodes were inserted subcutaneously in the right forearm and left hind leg, lead II electrocardiogram was recorded and data was analyzed using LabChart 8 software (ADInstruments, New Zealand).

Cardiomyocyte Isolation

Mouse ventricular cardiomyocytes were enzymatically isolated. Briefly, mice were heparinized, anesthetized with 5% isoflurane, hearts were excised by thoracotomy, cannulated at the aorta, mounted on Langendorff perfusion apparatus and retrogradely perfused with Ca^{2+}-free Tyrode solution (containing in mM: 140 NaCl, 5.4 KCl, 2.5 $MgCl_2$, 5.5 HEPES and 11 glucose; pH 7.6) at 37°C for 4 min to washout blood remains. Then, hearts were perfused with the same solution containing type II collagenase (1 mg/mL, Worthington Biomedical) and type XIV protease (0.1 mg/mL, Sigma-Aldrich) at 37°C for 11 min to enzymatically digest the heart tissue. Subsequently, atria were cut off, digested ventricular tissue were teased apart under Tyrode solution (37°C) using forceps, followed by gentle mechanical agitation using small dropper pipette and filtered through a 100 µm mesh into a beaker to obtained single cells. Afterward, the Isolated cardiomyocytes were reconstituted in Ca^{2+}-containing external solution appropriate for each study, stored at room temperature (20-22°C) and used for experiments within 6 h after isolation.

Single-Cell Patch Clamp

Isolated ventricular myocytes were placed in a flow-through optical chamber mounted on the stage of an Olympus IX73 inverted microscope (Olympus Corporation, Tokyo, Japan), superfused with external solution appropriate for each recording and maintained at 37°C throughout the experiments. Whole-cell voltage clamp and current clamp recordings of membrane currents and voltage were obtained using a MultiClamp 700B amplifier (Molecular

Devices, Sunnyvale, CA, USA) connected to a computer by an Axon Digidata 1550B converter (Molecular Devices, Sunnyvale, CA, USA) and controlled by pClamp software (version 11.2; Molecular Devices). Patch pipettes were pulled from borosilicate glass (BF165-120-7.5, Sutter Instrument, Novato, CA, USA) using PC-100 pipette puller (Narishige, Tokyo, Japan). Pipettes had an electrical resistance of 0.85 to 1.5 MΩ when filled with internal solution appropriate for each recording.

To record action potential (AP), cardiomyocytes were maintained in external solution containing (in mM): 124 NaCl, 25 $NaHCO_3$, 1.2 $CaCl_2$, 4 KCl, 1 $MgCl_2$, 10 HEPES, and 10 glucose (pH adjusted to 7.6 with NaOH). Internal solution contained (in mM): 130 L-aspartic acid potassium salt, 25 KCl, 5 NaCl, 3 Mg-ATP, 10 HEPES, 10 Phosphocreatine, 0.01 EGTA, 0.002 cAMP (pH adjusted to 7.2 with KOH). APs were evoked by injecting current (1 - 3 nA) with a pulse width of 2 - 3 ms at a frequency of 1 Hz.

To record L-type calcium current ($I_{Ca,L}$), cells were maintained in external solution contained (in mM): 137 NaCl, 5.4 CsCl, 0.5 $MgCl_2$, 1 $CaCl_2$, 0.33 NaH_2PO_4, 5 HEPES, 5.5 glucose (pH adjusted to 7.6 with NaOH). The internal solution contained (in mM): 130 CsCl, 20 TEA-Cl, 5 EGTA, 5 Mg-ATP, 5 HEPES, and 0.06 Tris-GTP (pH adjusted to 7.2 with CsOH). In both solutions, K was replaced with Cs to eliminate interference of voltage dependent K channels. Holding potential was set at −80 mV, pre-pulse was applied to −40 mV lasting 50 ms (to block fast Na current, I_{Na}) and $I_{Ca,L}$ was evoked by 150 ms voltage steps from a test potential of −40 mV to +50 mV in 10 mV increments.

To record total outward potassium current ($I_{K\text{-total}}$), cells were maintained in external solution contained (in mM): 140 NaCl, 4 KCl, 1 $MgCl_2$, 2 $CaCl_2$, 5 HEPES, 10 glucose, and 0.002 nifedipine (pH adjusted to 7.4 with NaOH). Internal solution contained (in mM): 135 KCl, 2 $MgCl_2$, 10 HEPES, 5 EGTA, 2 Mg-ATP, (pH adjusted to 7.2 with KOH). Holding potential was set

at -80 mV, $I_{K\text{-}total}$ was evoked by voltage steps from test potential of −60 mV to +50 mV in 10 mV increments lasting 3 s.

Transient outward potassium current (I_{to}), slowly inactivating potassium current ($I_{K,slow}$), and steady-state potassium current (I_{ss}) were isolated from the $I_{K\text{-}total}$ using previously described and validated biophysical and pharmacological approaches (2–4). Briefly, I_{to} was separated from the total outward current using an inactivating prepulse protocol. $I_{K,slow}$ was subsequently isolated from the remaining current by applying a low concentration (100 μM) of 4-aminopyridine (4-AP). The residual current remaining after elimination of both I_{to} and $I_{K,slow}$ was quantified as I_{ss}.

Statistics

Statistical analyses were performed using GraphPad Prism 10 (GraphPad Software). Data are presented as mean ± SD unless otherwise indicated. Comparisons between two groups were performed using two-tailed Student's *t* test or the Mann-Whitney test, as appropriate. Comparisons among multiple groups were performed using nested one-way ANOVA, ordinary two-way ANOVA, mixed-effects analysis, or the Kruskal-Wallis test, as appropriate. For cardiomyocyte experiments, cardiomyocytes were nested within mice, with mice treated as the experimental unit. A two-sided P value < 0.05 was considered statistically significant.

Study Approval

The study was approved by the University of California, Los Angeles (UCLA) Institutional Animal Care and Use Committee (protocol #: R-18-046). All animal experiments complied with institutional guidelines and ethical regulations, and adhered to the Guide for the Care and Use of Laboratory Animals of the Institute of Laboratory Animal Research, National Research Council (US).

Data availability

The values underlying all graphs and summary statistics reported in this manuscript are provided in the **Supporting Data Values** file. Additional data supporting the findings of this study are available from the corresponding author upon reasonable request.

Author Contributions

SEE performed experiments, analyzed data, and wrote the manuscript. CM performed experiments and analyzed data. JP and ZZ performed computational modeling and analyzed data. HH provided the NPY mouse model and edited the manuscript. ZQ performed computational modeling and edited the manuscript. KS, RH, and OAA contributed to study design, supervised the study, and edited the manuscript. All authors reviewed and approved the final manuscript.

References

1. Karl T, Duffy L, Herzog H. Behavioural profile of a new mouse model for NPY deficiency. *European Journal of Neuroscience*. 2008;28(1):173–180.

2. Xu H, Guo W, Nerbonne JM. *Four Kinetically Distinct Depolarization-activated K Currents in Adult Mouse Ventricular Myocytes*. 1999.

3. Liu J, et al. Dissection of the voltage-activated potassium outward currents in adult mouse ventricular myocytes: Ito,f, Ito,s, I K,slow1, IK,slow2, and Iss. *Basic Res Cardiol*. 2011;106(2):189–204.

4. Brouillette J, et al. Functional properties of K+ currents in adult mouse ventricular myocytes. *Journal of Physiology*. 2004;559(3):777–798.

SUPPLEMENTAL FIGURE 1

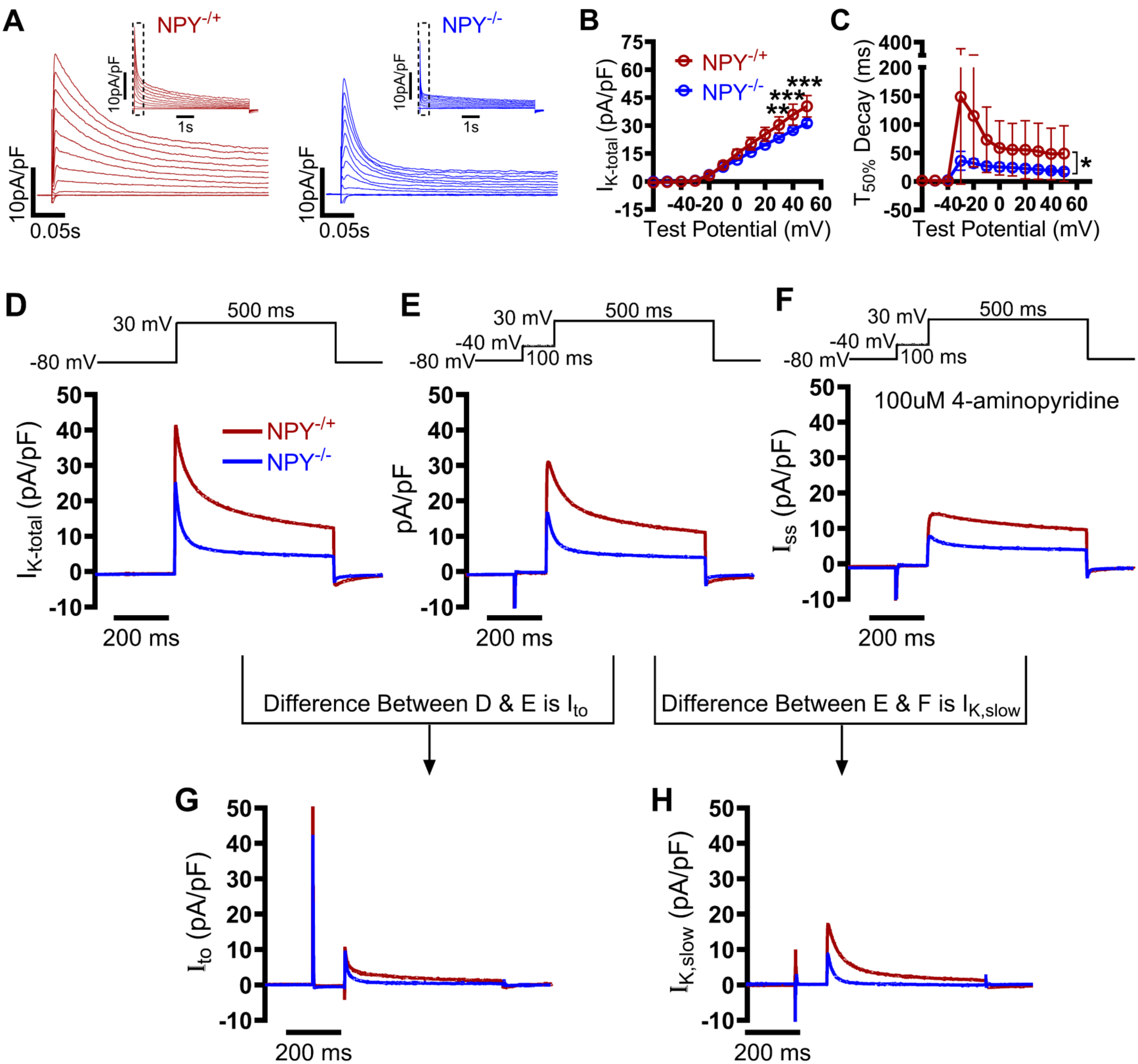

Supplemental Figure 1. NPY depletion alters outward K^+ current properties in ventricular cardiomyocytes. (A) Representative traces of total outward potassium current ($I_{K\text{-total}}$) recorded at varying test potentials in $NPY^{-/+}$ and $NPY^{-/-}$ ventricular cardiomyocytes. **(B)** Current-voltage relationship plot of $I_{K\text{-total}}$. **(C)** Time to ½ inactivation of $I_{K\text{-total}}$. **(D)** Representative traces of $I_{K\text{-total}}$ recorded in both groups using the voltage-clamp protocol shown above the traces. Cells were depolarized from a holding potential of −80 mV to +30 mV for 500 ms. **(E)** Representative current traces recorded using an inactivating prepulse protocol (−40 mV for 100 ms) to inactivate transient outward potassium current (I_{to}) before the test pulse to +30 mV. Under these conditions, the remaining current represents $I_{K\text{-total}}$ without I_{to}. **(F)** Representative traces recorded in the presence of 100 µM 4-aminopyridine (4-AP) to inhibit slowly inactivating potassium current ($I_{K,slow}$). The residual current under these conditions represents the steady-state potassium current (I_{ss}). **(G)** I_{to} obtained by digital subtraction of currents in panel E from those in D (D − E). I_{to} density was comparable between $NPY^{-/+}$ and $NPY^{-/-}$ cardiomyocytes. **(H)** $I_{K,slow}$ obtained by subtracting currents in panel **F** from those in **E** (E − F). $I_{K,slow}$ was reduced in $NPY^{-/-}$ cardiomyocytes compared with $NPY^{-/+}$ control. Currents are normalized to cell capacitance and expressed as current density (pA/pF). Data are mean ± SD, with cardiomyocytes nested within mice and mice treated as the experimental unit; *$p < 0.05$, **$p < 0.01$, ****$p < 0.0001$.

SUPPLEMENTAL FIGURE 2

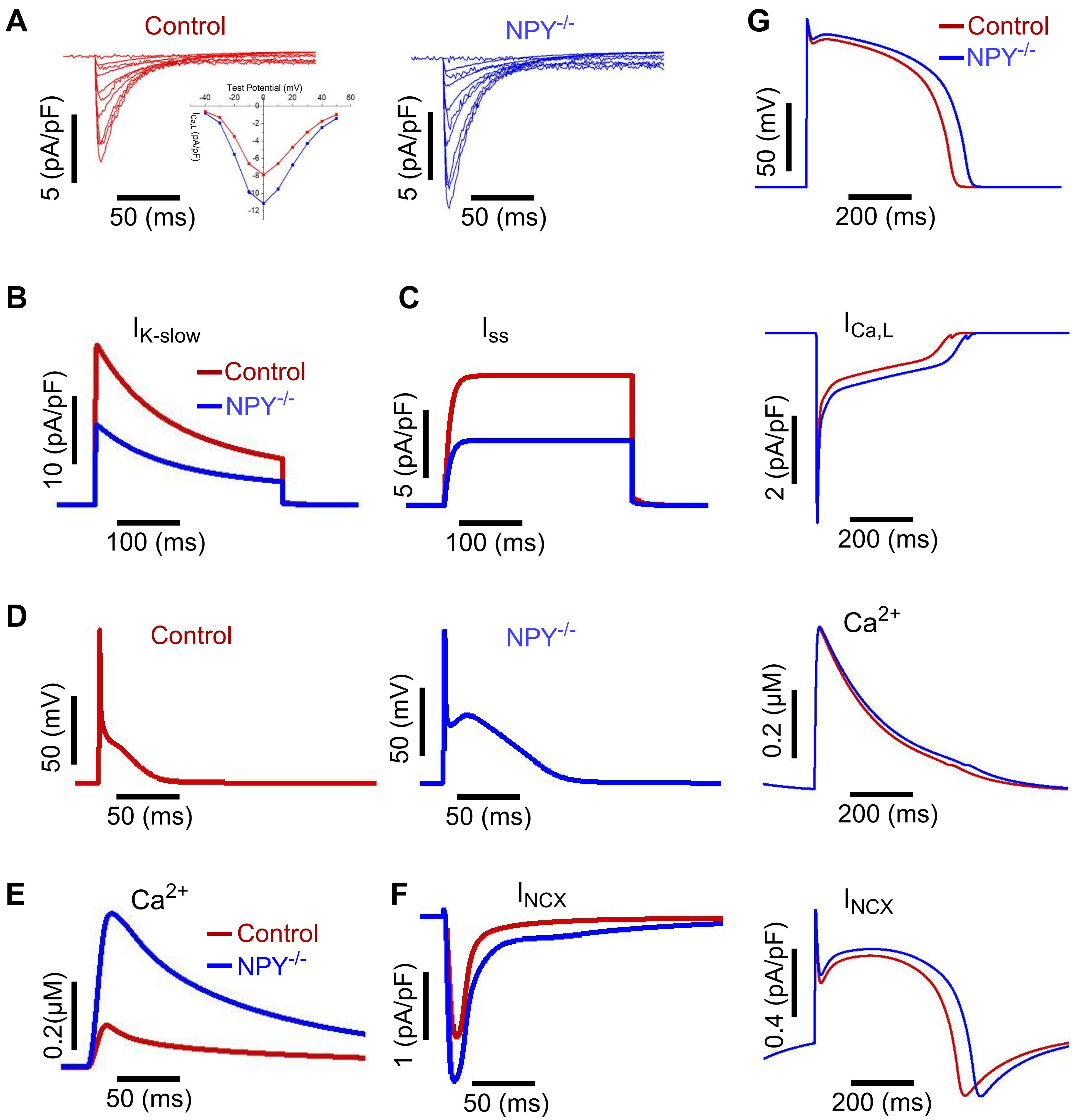

Supplemental Figure 2. Computational modeling of altered $I_{Ca,L}$, $I_{K,slow}$, and I_{ss} reproduces AP changes in NPY$^{-/-}$ cardiomyocytes and predicts corresponding effects in human ventricular myocytes. (A) simulated $I_{Ca,L}$ in control and NPY$^{-/-}$ cardiomyocytes showing increased current density in NPY$^{-/-}$. **(B and C)** simulated $I_{K,slow}$ and I_{ss} in control and NPY$^{-/-}$ cardiomyocytes showing reduced current density in NPY$^{-/-}$. **(D)** simulated control and NPY$^{-/-}$ mice ventricular myocyte AP generated by 33% increase in $I_{Ca,L}$, 50% reduction in $I_{K,slow}$ and I_{ss} reproduce the APD prolongation observed experimentally. **(E)** intracellular Ca transient ([Ca]i) recorded from the simulated mice AP showing increased amplitude in NPY$^{-/-}$ myocytes. (F, sodium/calcium exchanger current (I_{NCX}) recorded from the simulated mice AP showing enhanced current in NPY$^{-/-}$ consistent with increased [Ca]i. **(G)** simulated effects of NPY depletion in human ventricular AP models generated by 40% increase in $I_{Ca,L}$ showing prolonged APD in endocardial cells with corresponding $I_{Ca,L}$, [Ca]i and I_{NCX}.

SUPPLEMENTAL FIGURE 3

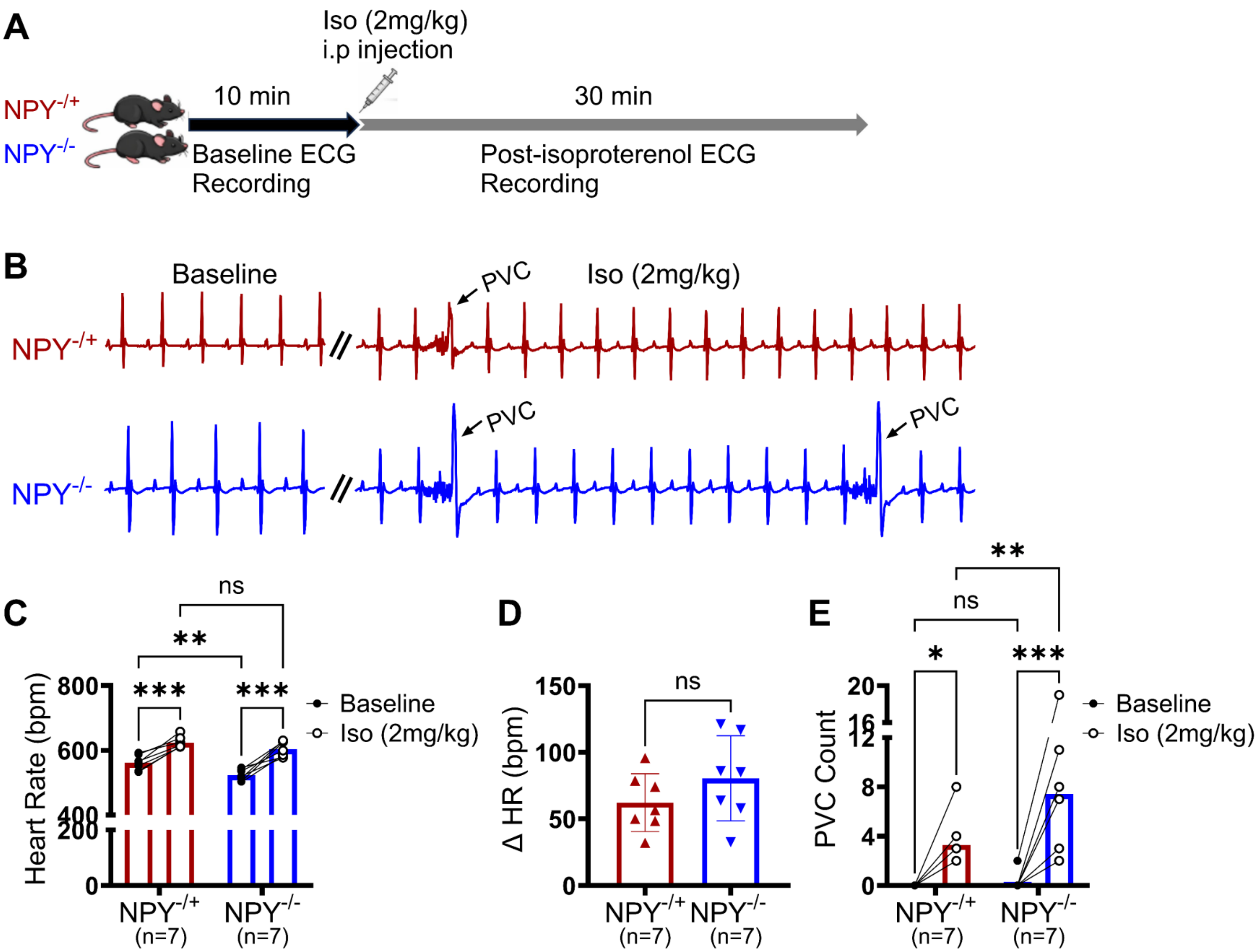

Supplemental Figure 3. NPY deficiency increases susceptibility to isoproterenol-induced ventricular ectopy. (A) Experimental protocol for in vivo electrocardiogram (ECG) recordings. Baseline ECGs were recorded for 10 min in mice followed by intraperitoneal injection of isoproterenol (Iso, 2 mg/kg) and ECG monitoring for an additional 30 min. **(B)** Representative ECG traces from NPY$^{-/+}$ and NPY$^{-/-}$ mice at baseline and after Iso administration. Premature ventricular complexes (PVCs) are indicated. **(C)** Quantification of heart rate at baseline and following Iso injection in NPY$^{-/+}$ and NPY$^{-/-}$ mice. **(D)** Change in heart rate (ΔHR) following Iso administration in NPY$^{-/+}$ and NPY$^{-/-}$ mice. **(E)** Quantification of PVC events observed during the recording period at baseline and after Iso injection. Iso induced significantly more PVCs in NPY$^{-/-}$ mice compared with NPY$^{-/+}$ mice. Data are mean ± SD; *p < 0.05, **p < 0.01, ***p < 0.001.

SUPPLEMENTAL FIGURE 4

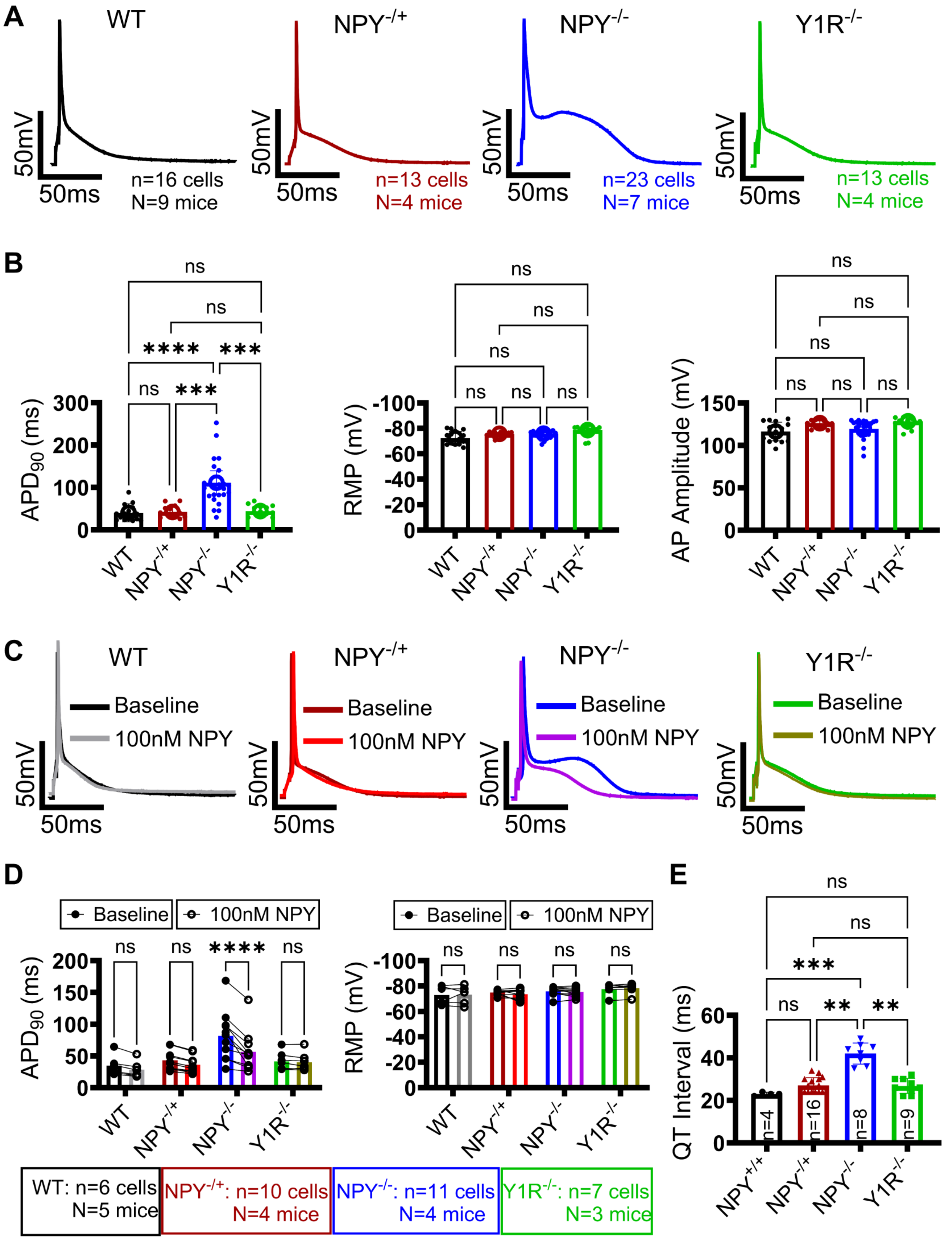

Supplemental Figure 4. NPY depletion-induced ventricular repolarization abnormalities are independent of Y1 receptor (Y1R) signaling. (A) Representative ventricular cardiomyocyte action potentials recorded from WT, $NPY^{-/+}$, $NPY^{-/-}$, and $Y1R^{-/-}$ mice under baseline conditions. **(B)** Quantification of AP duration at 90% repolarization (APD_{90}), resting membrane potential (RMP), and AP amplitude. APD_{90} was prolonged in $NPY^{-/-}$ cardiomyocytes, whereas $Y1R^{-/-}$ cells exhibited values comparable to controls. **(C)** Representative action potentials before and after application of 100 nM NPY. **(D)** Paired analysis of APD_{90} and RMP before and after 100 nM NPY treatment. Exogeneous NPY significantly shortened APD in $NPY^{-/-}$ cardiomyocytes but not in $Y1R^{-/-}$ cells. **(E)** QT interval measurements from surface ECG recordings. Unlike $NPY^{-/-}$ mice, $Y1R^{-/-}$ mice showed QT intervals comparable to controls, indicating that ventricular electrophysiological abnormalities induced by NPY depletion are not mediated through Y1R signaling. Data are mean ± SD, with cardiomyocytes nested within mice and mice treated as the experimental unit; $^{**}p < 0.01$, $^{***}p < 0.001$, $^{****}p < 0.0001$.
